# Usage of Social Media and Smartphone Application in Assessment of Physical and Psychological Well-Being of Individuals in Times of a Major Air Pollution Crisis

**DOI:** 10.2196/mhealth.2827

**Published:** 2014-03-25

**Authors:** Melvyn WB Zhang, Cyrus SH Ho, Pan Fang, Yanxia Lu, Roger CM Ho

**Affiliations:** ^1^Southeast Asian Haze Research ConsortiumDepartment of Medical PsychologySchool of Medicine, Shandong UniversityChinaChina

**Keywords:** crisis, haze, Internet, Web-based medium, social networking, smartphone application

## Abstract

**Background:**

Crisis situations bring about many challenges to researchers, public institutions, and governments in collecting data and conducting research in affected individuals. Recent developments in Web-based and smartphone technologies have offered government and nongovernment organizations a new system to disseminate and acquire information. However, research into this area is still lacking. The current study focuses largely on how new social networking websites and, in particular, smartphone technologies could have helped in the acquisition of crucial research data from the general population during the recent 2013 Southeast Asian Haze. This crisis lasted only for 1 week, and is unlike other crisis where there are large-scale consequential after-effects.

**Objective:**

To determine whether respondents will make use of Internet, social media, and smartphone technologies to provide feedback regarding their physical and psychological wellbeing during a crisis, and if so, will these new mechanisms be as effective as conventional, technological, Internet-based website technologies.

**Methods:**

A Web-based database and a smartphone application were developed. Participants were recruited by snowball sampling. The participants were recruited either via a self-sponsored Facebook post featuring a direct link to the questionnaire on physical and psychological wellbeing and also a smartphone Web-based application; or via dissemination of the questionnaire link by emails, directed to the same group of participants. Information pertaining to physical and psychological wellbeing was collated.

**Results:**

A total of 298 respondents took part in the survey. Most of them were between the ages of 20 to 29 years and had a university education. More individuals preferred the option of accessing and providing feedback to a survey on physical and psychological wellbeing via direct access to a Web-based questionnaire. Statistical analysis showed that demographic variables like age, gender, and educational levels did not influence the mechanism of access. In addition, the participants reported a mean number of 4.03 physical symptoms (SD 2.6). The total Impact of Event Scale–Revised (IES-R) score was 18.47 (SD 11.69), which indicated that the study population did experience psychological stress but not post-traumatic stress disorder. The perceived dangerous Pollutant Standards Index (PSI) level and the number of physical symptoms were associated with higher IES-R Score (*P*<.05).

**Conclusions:**

This is one of the first few studies demonstrating the use of Internet in data collection during an air-pollution crisis. Our results demonstrated that the newer technological modalities have the potential to acquire data, similar to that of conventional technologies. Demographic variables did not influence the mechanism of usage. In addition, our findings also suggested that there are acute physical and psychological impacts on the population from an air-pollution crisis.

## Introduction

### Dissemination of Information

Crisis situations bring about many challenges to researchers, public institutions, and governments to collect data and conduct research in affected individuals. In every crisis, communication between the general population and government is the key to enhance survival and reduce harm. This is especially so given the unpredictable nature of the crisis, and when the impact resulting from the crisis is significant [1]. Citizens who are being implicated by the crisis are naturally highly aroused and stressed. Thus, public health organizations have an obligation to keep the general population adequately informed of the latest information, and to provide them with necessary advice based on the changes in the situation [2].

Past research emphasized that the most critical aspect during a crisis was the selection of appropriate communication channels that had the highest degree of coverage and to be able to reach the population at risk. Studies on a cohort of Americans identified that the most trustworthy source of information during a crisis came from the doctors [3]. Studies conducted on people in Southeast Asia or Malaysia during the recent H1N1 influenza crisis identified that newspaper, television, and family members were the most trustworthy sources of information [4]. In comparison, similar studies done in the European population, like the Dutch, identified that the most trustworthy source of information came from the mass media during an outbreak of respiratory infectious disease [5,6]. During the 2011 Enterohaemorrhagic *Escherichia coli* (EHEC) outbreak in Germany, the usage of a Web-based medium as a modality of communication of critical information was reported [7]. The study identified the Internet to be the most popular medium for dissemination of EHEC-related information, with news and newspaper websites as the most often consulted Web-based sources. Participants were noted to be skeptical of information posted on social networking sites and media like Facebook and Twitter [7], but this finding was preliminary due to small sample size (n=18 students).

### Case: The 2013 Southeast Asian Haze Crisis

Recently in Southeast Asia, the Southeast Asian Haze has been a major cause of concern for all of the Association of Southeast Asian Nations (ASEAN) countries, due to it being a major air pollution disaster, with significant implications on health, socioeconomic, and even politics. The countries usually affected include Malaysia, Singapore, Brunei, Southern Thailand, and Indonesia. The 2013 haze that occurred from the second half of June was reported to be one of the worst air-pollution crises in Southeast Asia with massive and far-reaching implications.

This large scale, repetitive, air-quality disaster dates back to the early 1900s, with the first serious episode occurring in 1997, when fires started in Kalimantan and Sumatra, Indonesia by farmers who adopted the “slash-and-burn” technique of clearing land for agricultural usage. The estimated economic loss caused by the 1997 haze crisis was estimated to be US $9 billion. In consideration of the massive impact of haze disaster, the ASEAN Haze Technical Task Force was established to implement regional and National Haze Action Plans, as well as strict no-burn policy in ASEAN countries. Despite efforts being taken to avoid future haze disaster, there was a recurrence in 2006.  Economists estimated that Singapore lost at least US $50 million during the 2006 haze crisis. Industries like construction (7% of GDP) and tourism (3% of GDP) were severely affected. In addition, the losses were attributed to increase in health care costs, as well as reduction in productivity as a result of sick leave and inefficiency.

Unlike 1997 and 2006, the 2013 haze crisis has led to devastating effects for Indonesia’s neighboring countries, such as Singapore and Malaysia. In view of the poor air quality, the Singapore government has taken measures to safeguard the physical wellbeing of Singaporeans [8]. Even in healthy individuals, the haze may cause medical complications like conjunctivitis, throat irritation, rhinitis, blocked nasal passages, excessive sputum production, and even significant breathlessness, headache, and slow cognition. In addition, those who have preexisting respiratory conditions may find their conditions worsening; those with dermatological conditions like eczema might also find exacerbation of their skin conditions; those with preexisting cardiovascular conditions may suffer an increased risk of myocardial infarction or stroke; and those who are pregnant and are asthmatic may have reduced oxygenation of the fetus, thus affecting fetal growth [8]. The acute onset of such a massive air pollution crisis was what motivated the authors to consider whether conventional technologies, as described in previous research, and newer modalities of technology would be appropriate in reaching out to the general population, in terms of dissemination of necessary crisis information, as well as collation of crucial information from the general population.

Previous research has highlighted the usage of the Internet and in a crisis situation. The recent developments in Web-based technologies such as Facebook and Twitter, have offered government and nongovernment organizations with new means of disseminating vital information. These new technological modalities have the potential to acquire information and feedback from the general population during a crisis as well, which is important in assessing how the population is coping and responding thus far to the crisis. In particular, Keim and Noji [9] stated that the usage of social media initially started out during the 2010 Haiti earthquake, where various technological modalities such as text messaging, Facebook, and other social media were used in the immediate aftermath of the earthquake. Text messages were used as a means to gather donations and funds for relief work. Other technological modalities were used to disseminate information, gather donations, and also provide psychological support.  Previous research has also been done using Facebook technologies to compare the levels of post-traumatic stress symptoms that individuals experienced during the 2011 Fukushima nuclear disaster [10]. These allow the government and relevant agencies to closely monitor the current situation at the ground level and adjust the level of support accordingly [11]. Information acquired from the general population includes physical and physiological symptoms, as well as detecting new cluster of outbreaks of infectious diseases [11].

Apart from the developments in Web-based technologies, there have been further advances in mobile phone technologies, with the increasing popularity of smartphones. Smartphones are equipped with immense computing capabilities and allow individuals to access the Internet on the go. There has been a massive surge in the number of smartphone applications that are made available for downloading. Statistics show an increase from 300 million applications being downloaded in 2009 to over 5 billion in 2010 [12]. In particular, there are 7000 health care-related applications available as of 2011 [13]. Most of these health care applications are able to provide information, advice, instructions, and have various other interactive tools for individuals to monitor, record, and reflect their physical and psychological wellbeing [13].  This application is valuable as the uncertainty and lack of accurate information often lead to psychological distress.

Research looking into how newer Web-based technologies, such as the Internet, advertising on social media like Facebook, and, in particular, the latest technologies, such as smartphone applications, in acquiring crucial information during a crisis is limited. Hence, our study focuses largely on how existing social networking sites like Facebook and newer smartphone technologies could help in the acquisition of crucial research data from the general population during a crisis.  We applied our latest technology in the recent 2013 Southeast Asian Haze Crisis. The results that we obtained would be informative in helping researchers, public organizations, and governments to assess and gather information from the general population rapidly during a major crisis. Our research objectives were: did the general population use Internet technologies like social media and smartphone technologies to provide feedback to agencies regarding their physical and psychological well-being during an air pollution crisis, and if so, will these newer mechanisms (social media and smartphone) be as effective as conventional Internet-based website technological modalities?

## Methods

### Database Development

The questionnaire (Multimedia Appendix 1), which included (1) demographics, (2) physical symptoms experienced during haze, (3) perspectives on usefulness of health care equipment, and (4) Impact of Event Scale–Revised (IES-R) was written in English and was included in both the Facebook direct link as well as in our Web-based, mobile smartphone application. The Web-based questionnaire being featured was coded using a website (Polldaddy). This Web-based database development software was specially chosen, as it is one of the most robust platforms on the market that enables the capture of detailed information, such as the mechanisms of accessing the database (whether via a direct link or from a mobile device) and other pertinent information that is relevant to our current study. The demographics questionnaire comprised of seven items in total, and was used to acquire the baseline characteristics of the participants in the study, such as gender, age, ethnicity, marital status, level of education, occupation, and most importantly, presence or absence of chronic medical illnesses. The questionnaire on physical symptoms assessed the presence or absence of the following physical symptoms: mental slowing, headache, dizziness, eye discomfort, nose discomfort, mouth and throat discomfort, breathing difficulty, heart or chest pain, nausea and vomiting, gastric or abdominal discomfort, slowness in movement, and muscle ache or pain. The participants rated on the range of self-perceived dangerous Pollutant Standards Index (PSI) values, personal possession and perceived usefulness of the N-95 mask on 5-point Likert scales.  The IES-R is a 22-item, self-administered questionnaire that has been well validated for determining extent of stress reaction after exposure to stressful circumstances within 1 week of exposure across different cultural groups. Each item on the scale was administered via a 5-point frequency scale (0=not at all, 1=a little bit, 2=moderately, 3=quite a bit, 4=extremely) and higher scores indicate higher level of psychological stress. The IES-R provides three subscores, the mean avoidance, intrusion, and hyperarousal scores, a total mean IES-R score (divided by the total number of items) and a total IES-R score (without division by the total number of items). A total IES-R score greater than or equal to 33 signifies the likely presence of post-traumatic stress disorder.

### Smartphone Development

**Figure 1:**
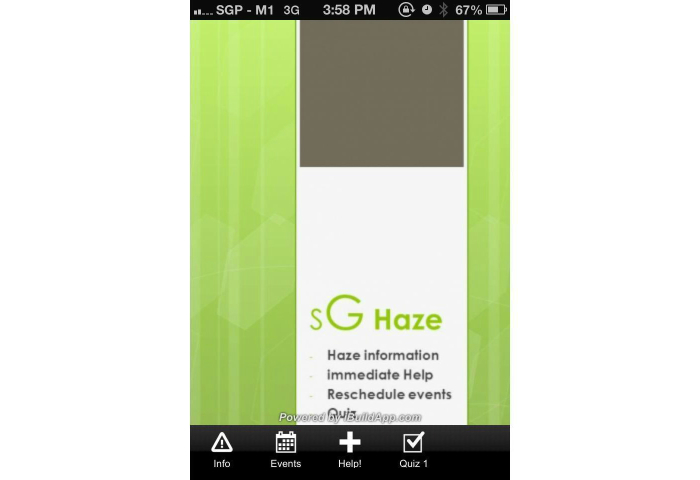
Overview of SgHaze smartphone application.

**Figure 2:**
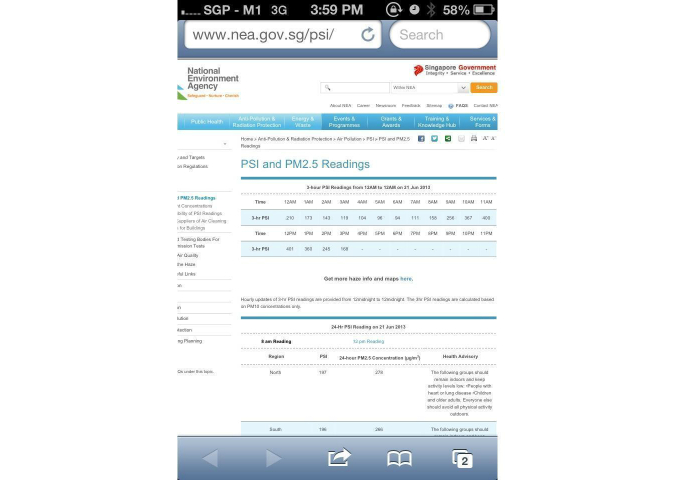
Pollutant Standard Index (PSI) information displayed within application.

**Figure 3:**
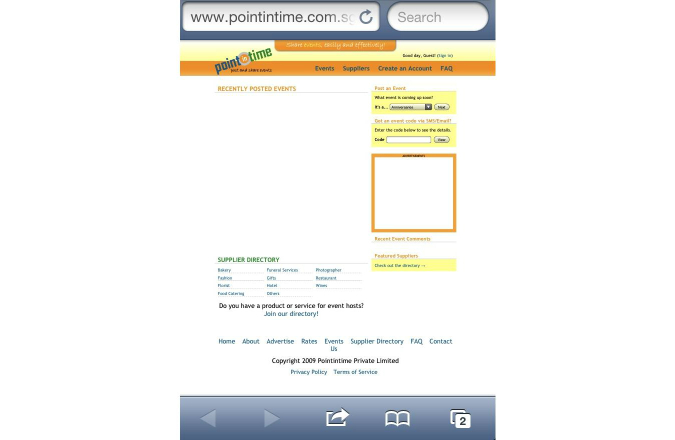
Event management system integrated within smartphone application.

**Figure 4:**
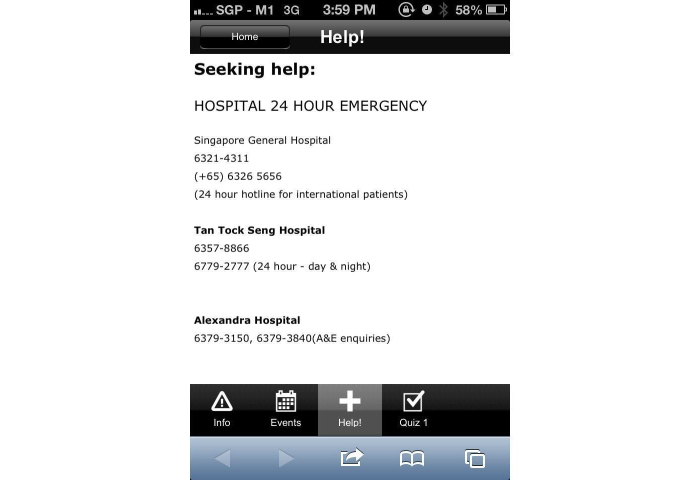
Emergency department and clinic contact details within application.

**Figure 5:**
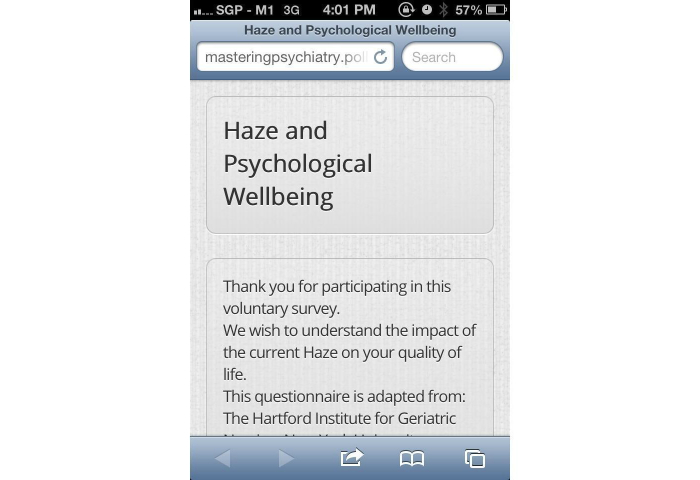
Web-based assessment quiz integrated within application.

**Figure 6:**
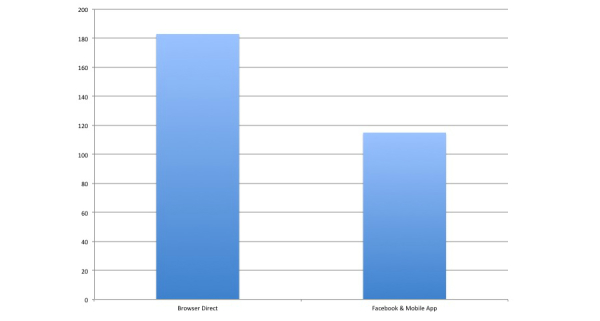
Comparison between direct Web-link access versus Facebook/smartphone access to Web-based questionnaire.

**Figure 7:**
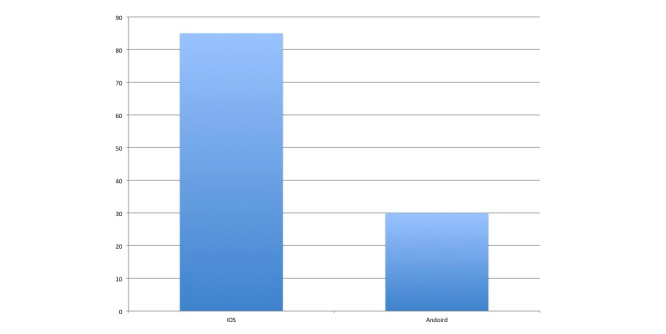
Overview of platform demographics of Facebook and smartphone application access.

In addition, a Web-based, smartphone-based application was also deployed. The Web-based smartphone application was designed to incorporate the following features: (1) general haze information, (2) events rescheduling system, (3) hospital emergency helplines and 24-hour clinic helplines and locations, and (4) a screening questionnaire, as previously mentioned. The smartphone application was developed to facilitate user’s accessibility of our questionnaire on their smartphone devices. The Web-based smartphone application was developed within hours of the commencement of the current air-quality crisis by means of a Web-based toolkit. The Web-based smartphone toolkit enabled the integration of the above-mentioned feature via basic hypertext markup language programming. The authors have developed a previous events rescheduling system that tapped in to a text messaging gateway to disseminate information, and this system was integrated within the application itself, as shown in Figures 1-7.

### Recruitment of Participants

This study was a cross-sectional study and participants were recruited by snowball sampling. The sampling was done either via a self-sponsored Facebook post that featured a direct link to the questionnaire and also the smartphone Web-based application; or via dissemination of the questionnaire link by emails, directed to the same group of participants (participants within the network circle of friends of all the authors, and there were opportunities for interaction between participants on the social network if they were common friends to the authors). The survey was conducted between the periods of June 21st and June 26th, 2013. The end date of recruitment was decided upon as the haze crisis had improved significantly with the air quality in the healthy range by the June 26th, 2013.

Participants who were either the friends of the authors or medical students previously under the supervision of the authors were invited to participate in the Web-based database survey. They needed to fulfill several selection criteria.

These included participants who were (1) above the age of 11-years old, (2) able to comprehend English, (3) living in Singapore during the period of the crisis, (4) had been exposed to the environmental air, and (5) knew how to use technological equipment.

Ethics approval was sought by the members of the Southeast Asian Haze Research Consortium from the institutional review board of the Department of Medicine, Shandong University, People’s Republic of China. All participants provided their consent online prior to commencement of the study.

### Analysis

The dataset was first extracted from the database and then it was coded. The demographics data were subsequently analyzed using Microsoft Excel, 2013. Basic statistics for each categorical variable, which was matched against the methodology of accessing the database, was computed. Additional statistical analysis involving the other questionnaires was performed using the SPSS statistical program, version 16.0 for Windows. Categorical variables were expressed by number and percentage. Continuous variables were expressed as mean (SD).  Chi-square analyses were used to compare demographic data, personal views on the N-95 mask, and presence of physical symptoms across two groups based on gender and perceived dangerous PSI value. Using a subscale of IES-R and total IES-R score as dependent variables, univariate linear regression were performed to identify factors, which determined psychological impact during the haze crisis. Statistical significance was set at *P*<.05 for all analyses.

## Results

A total of 298 individuals participated in this Web-based survey. Table 1 summarizes the basic demographic characteristics of all the 298 respondents. Most of the respondents in the current study were between the ages of 11 and 29 years, and the majority were Chinese (210/298, 70.5%). In addition, the majority of the respondents had an undergraduate education.

Table 2 summarizes the demographic characteristics of the participants who took part in our Web-based survey either via a direct link to the Web-based questionnaire, or via our Facebook-sponsored post and smartphone application. The demographics data captured by our Web-based database (Polldaddy) did not manage to distinguish between users who have accessed the Web-based questionnaire via Facebook or via Smartphone application. Most of the respondents in the current study (112/183, 61.4%) selected the option of accessing the questionnaire directly via the Web link that was emailed to them. Only 38.5% (44/115) of the respondents accessed the questionnaire toolkit via our Facebook post or via our smartphone Web-based application, which showed quantitatively that newer mechanisms of access were less popular. Chi-square statistical analysis was conducted to determine whether demographic variables (gender, educational status, and age) were indeed significant predictors influencing the mechanism of access (direct vs Facebook or smartphone application). Comparisons between the two groups were done and demographic variables, such as gender (χ^2^_1_=0.101, *P*=.809), educational status (χ^2^_1_=0.82, *P*=.413), and age (divided into 2 groups: younger than 29, and older than 30) (χ^2^_2_=2.33, *P*=.311) were found to be insignificant in influencing mechanism of access.

**Table 1:** Demographic characteristic of respondents (N=298)

Characteristic		Total sample (N=298)	Men (n=120)	Women (n=178)
		n (%)	n (%)	n (%)
**Age**				
	11-29	210 (70.5)	82 (68.3)	82 (26.3)
	30-69	88 (29.5)	38 (31.7)	38 (31.7)
**Race**				
	Chinese	260 (87.2)	99 (82.5)	161 (90.4)
	Others: Indians, Malays, and Others	38 (12.8)	21 (17.5)	17 (9.6)
**Educational status**				
	Undergraduate	251 (84.2)	100 (83.3)	152 (85.3)
	Others	47 (15.8)	20 (16.7)	26 (14.7)

**Table 2:** Demographic characteristics of respondents who accessed the questionnaire directly compared with those who accessed via Facebook or smartphone.

Characteristic		Direct link sample size	Facebook/smartphone sample size
		n (%)	n (%)
**Gender**			
	Men	80 (43.7)	40 (34.8)
	Women	103 (56.2)	75 (65.2)
**Age**			
	10-19 years	10 (5.5)	2 (1.7)
	20-29 years	114 (62.2)	83 (72.2)
	30-39 years	36 (19.8)	17 (14.9)
	40-49 years	10 (5.5)	9 (7.8)
	50-59 years	11 (6.0)	2 (1.7)
	60-69 years	1 (0.5)	2 (1.7)
	Older than 69 years	1 (0.5)	0 (0)
**Race**			
	Chinese	154 (84.2)	106 (92.3)
	Indian	17 (9.3)	2 (1.7)
	Caucasian	3 (1.6)	1 (0.8)
	Malays	5 (2.7)	3 (2.6)
	Others	4 (2.2)	3 (2.6)
**Education**			
	University	152 (83.1)	100 (87)
	Junior college	22 (12.0)	12 (10.5)
	Secondary school	9 (4.9)	2 (1.7)
	No education	0 (0)	1 (0.8)

In addition, the respondents reported a mean number of 4.03 physical symptoms (SD 2.6) and the total IES-R score was 18.47 (SD 11.69). The five most common physical symptoms among the 298 participants included mouth or throat discomfort (205/298, 68.8%), nose discomfort (183/298, 61.4%), eye discomfort (181/298, 60.7%), headache (149/298, 50.3%), and breathing difficulty (120/298, 40.3%). For the psychological impact, the mean intrusion score (mean 0.96, SD 0.63) was the highest, followed by mean hyperarousal score (mean 0.85, SD 0.74), and the mean avoidance score (mean 0.71, SD 0.5) was the lowest. When comparing the responses between male and female participants, 97.7% of the women were more likely to report usefulness of the N-95 mask (χ^2^_1_=7.353, *P*=.007) and 66.3% of the women also reported the presence of eye discomfort (χ^2^_1_=5.718, *P*=.017). There were no significant differences between men and women in demographics variables and scores on psychological impact. Respondents were further classified into two groups based on the perceived dangerous PSI value. Respondents who perceived lower PSI value (<250) as dangerous were more likely to come from other ethnic groups (χ^2^_1_=9.487, *P*=.002); report the presence of mouth or throat discomfort (χ^2^_1_=10.236, *P*=.001), nausea or vomiting (χ^2^_1_= 5.697, *P*=.017), higher number of physical symptoms (*t*_*296*_=2.522, *P*=.012), higher mean intrusion score (*t*_*296*_= 2.198, *P*=.029), higher mean hyperarousal score (*t*_*296*_=2.488, *P*=.013), higher total mean IES-R score (*t*_*296*_=1.990, *P*=.047), and higher total IES-R score (*t*_*296*_=1.990, *P*=.047).

Table 3 in Multimedia Appendix 2 summarizes the factors that determined the psychological impact during the haze crisis. The perceived dangerous PSI level was negatively associated with the mean intrusion score, where B is the effect size and SE is the standard error (B=-0.162, SE=0.074, *R* ^2^=.016, *P*=.029), mean hyperarousal score (B=-0.217, SE=0.087, *R* ^2^=.020, *P*=.013), total mean IES-R score (B=-0.124, SE0.062, *R* ^2^=.013, *P*=.047), and total IES-R score (B=-2.734, SE=1.374, *R* ^2^=.013, *P*=.047). The total number of physical symptoms was positively associated with the mean avoidance score (B=0.048, SE=0.011, *R* ^2^=.061, *P*<.001), mean intrusion score (B=0.075, SE=0.013, *R* ^2^=.095, *P*<.001), mean hyperarousal score (B=0.130, SE=0.015, *R* ^2^=.207, *P*<.001), total mean IES-R score (B=0.080, SE=0.011, *R* ^2^=.153, *P*<.001), and total IES-R score (B=1.759, SE=0.240, *R* ^2^=.153, *P*<.001). The demographic variables, actual PSI score during the survey and views on the N-95 masks were not associated with psychological impact during the haze crisis (*P*>.05).

## Discussion

### Principal Findings

From our current knowledge, this is one of the few studies to formally assess the application of Internet-based social media and a smartphone-based application to gather data from the general population during a crisis. This study was methodologically feasible and demonstrated the usefulness of social medial and a smartphone-based application during a severe air-pollution crisis, which necessitated individuals to stay indoors, and helped to reduce the barriers pertaining to recruitment. Multiple social activities and events were curtailed, and the general public had to engage in other home-based activities. Engaging in Web-based social networking activities was a popular option. Our current findings showed that more individuals preferred the option of accessing and providing feedback to a questionnaire on physical and psychological well-being by direct Internet Web-link access, as compared with indirect access via Facebook or even a smartphone application. Our results did show, that for our current study, demographic variables such as gender, level of education, and age did not significantly affect the mechanism of access. Our current study showed that for the Asian population surveyed, demographic variables did not predict whether individuals would use either direct Internet access to provide feedback about their physical and psychological well-being, or whether they would use newer modalities of technologies like social media (Facebook) or a smartphone application.

In addition, our findings show that the perceived dangerous PSI value, not the actual PSI value and number of physical symptoms determined the psychological impact during the haze crisis. Furthermore, the higher number of physical symptoms was associated with greater psychological impact. This finding suggests that reduction in the number of physical symptoms may reduce psychological impact during haze crisis. Also, from our analysis, the total IES-R score was 18.47 (SD 11.69). A total IES-R score of 33 or more signifies the likely presence of post-traumatic stress disorder [14]. Our results suggest that the haze did not cause post-traumatic stress disorder in the general population, but it caused mild to moderate psychological impact. Intrusive symptoms such as recurrent thoughts and feelings were more common than hyperarousal and avoidance symptoms in our study population.  The local government played a key role in reducing the psychological impact. On June 21, the local government took immediate measures to protect its citizens, including hourly updates of the PSI value on television, cancellation of all school activities, offering medical subsidies to treat physical discomfort associated with haze, and providing 1 million free N-95 masks to the needy households [15]. As a result, the personal possession and perceived usefulness of N-95 masks were not predictors of psychological stress.

As there was no previous study on the physical and psychological impact of the haze crisis, the interpretation of our findings mainly relied on previous studies on infectious disease outbreak, such as severe acute respiratory syndrome (SARS) in the Southeast Asia. Our findings were similar to a study reported by Lee et al [16] on the psychological responses of pregnant women during the SARS outbreak in Hong Kong in 2003. Lee et al [16] reported that anticipatory worries were common among the pregnant women during the SARS outbreak; overestimation of risk led to higher level of anxiety and the levels of depression were similar between the SARS and pre-SARS outbreak cohorts. In our study, the inverse association between the perceived dangerous PSI values and mean scores of intrusion, hyperarousal, and overall psychological impact suggest that people who have lower threshold for health hazard are vulnerable to greater psychological impact when air quality deteriorates. The low threshold for health hazard and the number of physical symptoms independently determine psychological impact and these factors may contribute to the anticipatory anxiety. Our study did not find a difference in the levels of psychological impact between respondents who filled the questionnaire out during the periods with low and high PSI values. Lee et al [16] found that people tended to receive more social support during the SARS outbreak. We did not measure social support in our study but it may be possible that people would receive more social support from their families during the haze outbreak, as they tend to stay at home or participate in indoor activities with families.

### Strengths and Limitations

The main strength of the current study is that we managed to assess the feasibility of acquiring data from a general population using the latest information technology during a crisis. This study managed to evaluate the differences in mechanism of acquiring data and showed that demographic variables like gender, level of education, and age did not significant predict the mechanism of access to either conventional technological modalities or newer modalities like social medium and smartphones.

Nevertheless, there are several limitations in the current study. Our results are preliminary and cross-sectional in nature. The crisis that we have selected to evaluate lasted only for 1 week and participants were recruited over that short duration of time as the haze disappeared. Also, unlike that of other disasters, there was no large-scale consequential aftermath after the crisis, and most individuals were able to get back to normal life immediately, which is also a major limiting factor in our research.  In addition, our study has an inherent cohort bias, as the study population consisted of younger and educated individuals, recruited by snowball sampling, which is a nonrandom sampling method. Hence, even though we have identified the preferences of our respondents, the observation cannot be generalized to other populations. Nevertheless, we need to emphasize that we encountered major difficulties with rapid planning of this research, recruiting participants, and gathering data over such a short period of time with uncertainty. As the impact of the event lasted only 1 week, it is difficult to plan for and conduct a proper social media network and smartphone application survey.

### Conclusions

This is one of the few studies demonstrating the use of Internet in data collection during an air-pollution crisis. Our methods may apply to future research on a natural disaster or outbreak of an infectious disease. The Internet and recent advancements in information technology allow researchers to capture data during a crisis, which was impossible in the past. Traditional face-to-face interviews come to a halt when people have to be quarantined to avoid spread of infection or staying at home to avoid adverse physical environment. The Internet allows researchers to overcome such barriers. This study compared conventional Web-based methods of acquiring data against the newer methods, including the social networking sites and smartphone applications. Previous studies were limited to comparison of technologies in relation to dissemination of information.  Our results demonstrated that the newer technological modalities have the potential to acquire data as efficiently as conventional technologies, and demographic variables did not influence the mechanism of access during a crisis situation. Our current study provides an example for future researchers to develop a platform using the latest information technology to conduct research during any crisis. This is especially important for governments and health authorities to develop such platform in preparation for an unforeseen crisis.

In addition, the current study also highlights that even a short-term exposure to haze could potentially lead to considerable physical and psychological disturbances in healthy individuals. The findings provide guidance to health regulators to reduce the physical symptoms experienced. In addition, it is important to take note that it is the perceived PSI value that would determine psychological impact. Thus, timely governmental actions, which include increasing awareness and educating the general public, would help to reduce the psychological impact experienced.

## Multimedia Appendix 1

Sample questionnaire survey.

## Multimedia Appendix 2

Analysis of psychological impact of individuals affected by haze crisis.

## Abbreviations

ASEAN: Association of Southeast Asian Nations
B: effect size
EHEC: Enterohaemorrhagic Escherichia coli
IES-R: Impact of Event Scale–Revised
PSI: Pollutant Standards Index
SARS: severe acute respiratory syndrome
SE: standard error

## References

[1] Glik DC (2007). Risk communication for public health emergencies. Annu Rev Public Health.

[2] J. Holmes B, Henrich N, Hancock S, Lestou V (2009). Communicating with the public during health crises: experts' experiences and opinions. Journal of Risk Research.

[3] Avery E (2010). Contextual and audience moderators of channel selection and message reception of public health information in routine and crisis situations. J Public Relat Res.

[4] Wong LP, Sam IC (2010). Public sources of information and information needs for pandemic influenza A(H1N1). J Community Health.

[5] Bults M, Beaujean DJ, de Zwart O, Kok G, van Empelen P, van Steenbergen JE, Richardus JH, Voeten HA (2011). Perceived risk, anxiety, and behavioural responses of the general public during the early phase of the Influenza A (H1N1) pandemic in the Netherlands: results of three consecutive online surveys. BMC Public Health.

[6] Vartti AM, Oenema A, Schreck M, Uutela A, de Zwart O, Brug J, Aro AR (2009). SARS knowledge, perceptions, and behaviors: a comparison between Finns and the Dutch during the SARS outbreak in 2003. Int J Behav Med.

[7] van Velsen L, van Gemert-Pijnen JE, Beaujean DJ, Wentzel J, van Steenbergen JE (2012). Should health organizations use web 2.0 media in times of an infectious disease crisis? An in-depth qualitative study of citizens' information behavior during an EHEC outbreak. J Med Internet Res.

[8] National Environmental Agency.

[9] Keim ME, Noji E (2011). Emergent use of social media: a new age of opportunity for disaster resilience. Am J Disaster Med.

[10] Ben-Ezra M, Palgi Y, Aviel O, Dubiner Y, Soffer Y, Shrira A, Evelyn Baruch (2013). Face it: collecting mental health and disaster related data using Facebook vs. personal interview: the case of the 2011 Fukushima nuclear disaster. Psychiatry Res.

[11] Eysenbach G (2009). Infodemiology and infoveillance: framework for an emerging set of public health informatics methods to analyze search, communication and publication behavior on the Internet. J Med Internet Res.

[12] (2010). Mobile Future.

[13] Free C, Phillips G, Felix L, Galli L, Patel V, Edwards P (2010). The effectiveness of M-health technologies for improving health and health services: a systematic review protocol. BMC Res Notes.

[14] Weiss DS, Wilson JD, Tang C (2007). The Impact of Event Scale: Revised. Cross-cultural assessment of psychological trauma and PTSD.

[15] Ong A (2013). The Straits Times.

[16] Lee DTS, Sahota D, Leung TN, Yip ASK, Lee FFY, Chung TK (2006). Psychological responses of pregnant women to an infectious outbreak: a case-control study of the 2003 SARS outbreak in Hong Kong. J Psychosom Res.

