# Supplementary material for: Usage of Social Media and Smartphone Application in Assessment of Physical and Psychological Well-Being of Individuals in Times of a Major Air Pollution Crisis
Source: JMIR Mhealth Uhealth. 2014 Mar 25;2(1):e16. doi: 10.2196/mhealth.2827 (PMC4114481; doi:10.2196/mhealth.2827)
Supplement: Supplementary file 1 [file mhealth_v2i1e16_app1.pdf]

Haze and Psychological Wellbei...

Individual Participant

Participant Information

Location
Singapore
Unknown

Response ID	**48,951,068**
IP Address	**220.255.1.18**
Start Time	**15th Jul 2013 8:30 AM**
Finish Time	**15th Jul 2013 8:35 AM**
Type	**Mobile Device, Direct**
Platform	**Android 4**
Agent	**Android 4.0**

Question 01

A1. Gender

- Male
- ✓ **Female**

Question 02

A2. Age (in years)

- 10-19
- ✓ **20-29**
- 30-39
- 40-49
- 50-59
- 60-69
- older than 69

Question 03

A3. Ethnicity

- ✓ **Chinese**
- Indian
- Malay
- Eurasian
- Caucasian
- Others

Question 04

A4. Martial Status

- ✓ **Single (Heterosexual)**
- Single (Homosexual)
- Married
- Separated
- Divorced
- Window

Question 05

A5. Level of Education

No education

Primary School

✓ **Secondary School**

Junior College

University

Question **06**

Occupation

Housewife / home maker

Unemployed

Mainly involved outdoor work

Mainly involved indoor work

✓ **Working in healthcare industry**

Medical Student - First year

Medical Student - Second year

Medical Student - Third year

Medical Student - Fourth year

Medical student - Fifth year

Student - others

Question **07**

Do you have any of the undermentioned chronic illnesses?

Chronic lung disease

other chronic disease (e.g. diabetes)

✓ **NA. I have been in good health**

Question **08**

B1. Can you state the level of Pollutant Standard Index (PSI) which you find dangerous to your health (between 0 – 500)?

1. 0 – 49

2. 50 – 99

✓ **3. 100 – 149**

4. 150 – 199

5. 200 – 249

6. 250 – 299

7. 300 – 349

8. 350 – 399

9. 400 – 449

10. 450 – 499

11.> 500

Question **09** Do you experience the following symptoms? - Mental slowing

Yes
✓ **No**

Question **10** Do you experience the following symptoms? - Headache

✓ **Yes**
No

Question **11** Do you experience the following symptoms? - Dizziness

✓ **Yes**
No

Question **12** Do you experience the following symptoms? - Eye problem

✓ **Yes**
No

Question **13** Do you experience the following symptoms? - Nose problem

✓ **Yes**
No

Question **14** Do you experience the following symptoms? - Mouth and throat problem

Yes
✓ **No**

Question **15** Do you experience the following symptoms? - Breathing difficulty

✓ **Yes**
No

Question 16

Do you experience the following symptoms? - Heart pain or chest pain

Yes

✓ **No**

Question 17

Do you experience the following symptoms? - Nausea and vomiting

Yes

✓ **No**

Question 18

Do you experience the following symptoms? - Stomach and tummy discomfort

✓ **Yes**

No

Question 19

Do you experience the following symptoms? - Slowness in movement

Yes

✓ **No**

Question 20

Do you experience the following symptoms? - Muscle ache and pain

Yes

✓ **No**

Question 21

C1. How do you feel about availability of N-95 masks?

✓ **1.Very inadequate**

2.Inadequate

3.Available

4.Adequate

5.Very adequate

Question 22

C2. Do you feel that the N-95 mask is useful in protecting you against the haze?

1.Absolutely useless

2.Useless

✓ **3.Of some use**

4.Useful

5.Very useful

Question **23**

D1 Any reminder of haze situation (e.g. PSI score, news, picture of haze) brought back feelings about the haze.

0 = Not at all

1 = A little bit

✓ **2 = Moderately**

3 = Quite a bit

4 =Extremely

Question **24**

I had trouble staying asleep.

0 = Not at all

1 = A little bit

✓ **2 = Moderately**

3 = Quite a bit

4 =Extremely

Question **25**

Other things kept making me think about the haze situation in Singapore.

0 = Not at all

✓ **1 = A little bit**

2 = Moderately

3 = Quite a bit

4 =Extremely

Question **26**

I felt irritable and angry.

0 = Not at all

✓ **1 = A little bit**

2 = Moderately

3 = Quite a bit

4 =Extremely

Question **27**

I avoided letting myself get upset when I thought about the haze situation or was reminded of haze.

0 = Not at all

✓ **1 = A little bit**

2 = Moderately

3 = Quite a bit

4 =Extremely

Question **28** **I thought about the haze when I didn't mean to.**

0 = Not at all

✓ **1 = A little bit**

2 = Moderately

3 = Quite a bit

4 =Extremely

Question **29** **I felt as if the haze situation hadn't happened or wasn't real.**

0 = Not at all

✓ **1 = A little bit**

2 = Moderately

3 = Quite a bit

4 =Extremely

Question **30** **I stayed away from reminders about the haze.**

✓ **0 = Not at all**

1 = A little bit

2 = Moderately

3 = Quite a bit

4 =Extremely

Question **31** **Pictures about haze popped into my mind.**

0 = Not at all

✓ **1 = A little bit**

2 = Moderately

3 = Quite a bit

4 =Extremely

Question **32** **I was jumpy and easily startled.**

✓ **0 = Not at all**

1 = A little bit

2 = Moderately

3 = Quite a bit

4 =Extremely

Question **33** **I tried not to think about the haze situation in Singapore.**

0 = Not at all

✓ **1 = A little bit**

2 = Moderately

3 = Quite a bit

4 =Extremely

Question **34** **I was aware that I still had a lot of negative feelings about the haze, but I didn't deal with the feelings.**

✓ **0 = Not at all**

1 = A little bit

2 = Moderately

3 = Quite a bit

4 =Extremely

Question **35** **My feelings about the haze situation were kind of numb.**

✓ **0 = Not at all**

1 = A little bit

2 = Moderately

3 = Quite a bit

4 =Extremely

Question **36** **I always find myself acting or feeling like I was back at the time haze was very bad and PSI was very high.**

✓ **0 = Not at all**

1 = A little bit

2 = Moderately

3 = Quite a bit

4 =Extremely

Question **37** **I had trouble falling asleep.**

✓ **0 = Not at all**

1 = A little bit

2 = Moderately

3 = Quite a bit

4 =Extremely

Question **38**

I had waves of strong feelings about the haze.

✓ **0 = Not at all**

1 = A little bit

2 = Moderately

3 = Quite a bit

4 =Extremely

Question **39**

I tried to remove the haze situation from my memory.

✓ **0 = Not at all**

1 = A little bit

2 = Moderately

3 = Quite a bit

4 =Extremely

Question **40**

I had trouble concentrating.

✓ **0 = Not at all**

1 = A little bit

2 = Moderately

3 = Quite a bit

4 =Extremely

Question **41**

Reminders of the haze caused me to have physical reactions, such as sweating, trouble breathing, nausea, or a pounding heart.

0 = Not at all

✓ **1 = A little bit**

2 = Moderately

3 = Quite a bit

4 =Extremely

Question
42

I felt watchful and on guard about haze.

- ✓ **0 = Not at all**
- 1 = A little bit
- 2 = Moderately
- 3 = Quite a bit
- 4 =Extremely

Question
43

I tried not to talk about the haze.

- ✓ **0 = Not at all**
- 1 = A little bit
- 2 = Moderately
- 3 = Quite a bit
- 4 =Extremely
