# Supplementary material for: Usage of Social Media and Smartphone Application in Assessment of Physical and Psychological Well-Being of Individuals in Times of a Major Air Pollution Crisis
Source: JMIR Mhealth Uhealth. 2014 Mar 25;2(1):e16. doi: 10.2196/mhealth.2827 (PMC4114481; doi:10.2196/mhealth.2827)
Supplement: Supplementary file 2 [file mhealth_v2i1e16_app2.pdf]

Table 3. Demographic characteristics, perception of dangerous PSI level, and availability of N-95 masks, physical symptoms and psychological stress of the haze crisis (N = 298).

Item	Total sample (N=298)	Men (n=120)	Women (n=178)	*P* value	Perceived dangerous PSI[a] value < 250 (178)	Perceived dangerous PSI[a] value ≥250 (120)	*P* value
	N (%)	n (%)	n (%)		n (%)	n (%)	
**Age**							
18-29 (210)	210(70.5)	82(71.9)	128(68.3)	.507	126(71.2)	84(70.0)	.826
30-69 (88)	88(29.5)	38(28.1)	50(31.7)		51(28.8)	36(30.0)	
**Ethnicity**							
Chinese	260(87.2)	99(82.5)	161(90.4)	.044	164(92.1)	96(80.0)	.002
Indian, Malay, Other Ethnicities	38(12.8)	21(17.5)	17(9.6)		14(7.9)	24(20.0)	
**Martial Status**							
Married	71(23.8)	31(25.8)	40(22.5)	.297	39(21.9)	32(26.7)	.345
Others: Single, divorced, widow	227(76.2)	89(74.2)	138(77.5)		139(78.1)	88(73.3)	
**Occupation**							
Students	150(50.3)	65(54.2)	85(47.7)	.486	88(49.4)	62(51.7)	.893
Working Outdoor	66(22.2)	23(19.2)	43(24.2		41(23.1)	25(20.8)	
Working Indoor	82(27.5)	32(26.6)	50(28.1)		49(27.5)	33(27.5)	
**Presence of chronic medical**	20(7.3)	8(7.2)	12(7.4)	.950	13(8.0)	7(6.4)	.616

**illness**							
**Personal possession of N-95 mask**							
Inadequate –very inadequate	194(66.0)	72(61.0)	122(69.3)	.141	117(67.2)	77(64.2)	.584
Just enough – very adequate	100(34.0)	46(39.0)	54(30.7)		57(32.8)	43(35.8)	
**Physical symptoms**							
Mental slowing	82(27.5)	40(33.3)	42(23.6)	.065	53(29.8)	29(24.2)	.288
Headache	150(50.3)	90(50.6)	60(50.0)	.924	97(54.5)	53(44.2)	.080
Dizziness	74(24.8)	25(20.8)	49(27.5)	.190	51(28.7)	23(19.2)	.063
Eye discomfort	181(60.7)	63(52.5)	118(66.3)	.017	111(62.4)	70(58.3)	.485
Nose discomfort	191(64.1)	84(70.0)	107(60.1)	.081	122(68.5)	69(57.5)	.051
Mouth or throat discomfort	205(68.8)	78(65.0)	127(71.3)	.246	135(75.8)	70(58.3)	.001
Breathing difficulty	120(40.3)	42(35.0)	78(43.8)	.128	71(39.9)	49(40.8)	.870
Heart pain or chest pain	32(10.7)	16(9.0)	16(9.0)	.235	24(13.5)	8(6.7)	.062
Nausea or vomiting	30(10.1)	17(9.6)	13(10.8)	.718	24(13.5)	6(5.0)	.017
Gastric or abdominal discomfort	48(16.1)	16(13.3)	32(18.0)	.285	32(18.0)	16(13.3)	.285
Slowness in movement	51(17.1)	20(16.7)	31(17.4)	.866	34(19.1)	17(14.2)	.267
Muscle ache or pain	38(12.8)	15(12.5)	23(12.9)	.915	19(10.7)	19(15.8)	.190
Total number of physical symptoms	4.03(2.60)	3.93(2.74)	4.10(2.51)	.586	4.34(2.61)	3.58(2.53)	.012
**Psychological stress**							
Mean avoidance score mean ± SD	0.71(0.50)	0.70(0.52)	0.71(0.49)	.779	0.71(0.47)	0.70(0.55)	.779
Mean intrusion score mean ± SD	0.96(0.63)	0.94(0.62)	0.98(0.64)	.671	1.03(0.63)	0.87(0.61)	.029

Mean hyper-arousal score mean ±S D	0.85(0.74)	0.84(0.74)	0.86(0.75)	.889	0.94(0.73)	0.72(0.76)	.013
Total mean IES-R score mean± SD	0.84(0.53)	0.84(0.74)	0.85(0.53)	.740	0.89(0.51)	0.77(0.56)	.047
Total IES-R score mean ±(SD)	18.47(11.69)	18.19(11.73)	18.65(11.69)	0.740	19.57(11.18)	16.83(12.27)	0.047

[a]PSI = Pollutant Standard Index

Table 4. Univariate regression analysis of factors which determined the psychological impact (n = 298).

	**Mean avoidance score**				**Mean intrusion score**				**Mean hyper-arousal score**				**Total mean IES-R score**				**Total IES-R score**			
	B	SE	R^2	p-value	B	SE	R^2	p-value	B	SE	R^2	p-value	B	SE	R^2	p-value	B	SE	R^2	p-value
**Age**	0.028	0.064	0.001	0.663	-0.043	0.080	0.001	0.595	0.035	0.095	0	0.714	0.004	0.068	0	0.952	0.089	1.492	0	0.952
**Gender**	-0.017	0.059	0	0.779	-0.032	0.075	0.001	0.671	-0.012	0.088	0	0.889	-0.021	0.063	0	0.740	-0.460	1.383	0	0.740
**Ethnicity**	0.046	0.087	0.001	0.600	0.009	0.110	0	0.936	-0.031	0.130	0	0.810	0.011	0.092	0	0.902	0.250	2.033	0	0.902
**Marital status**	-0.004	0.068	0		0.092	0.086	0.004		0.064	0.101	0.001		0.050	0.072	0.002		1.093	1.591	0.002	

				0.958				0.284				0.526				0.493				0.493
**Occupation**	0.006	0.034	0	0.859	-0.009	0.043	0	0.837	0.036	0.051	0.002	0.480	0.009	0.036	0	0.809	0.193	0.796	0	0.809
**Presence of chronic medical illness**	0.097	0.118	0.003	0.410	-0.070	0.144	0.001	0.626	0.132	0.167	0.002	0.430	0.046	0.122	0.001	0.708	1.010	2.691	0.001	0.708
**Perceived dangerous PSI value**	-0.017	0.059	0	0.779	-0.162	0.074	0.016	0.029	-0.217	0.087	0.020	0.013	-0.124	0.062	0.013	0.047	-2.734	1.374	0.013	0.047
**The actual PSI value at the time of survey**	-0.001	0	0.012	0.055	0	0	0	0.907	0	0.001	0.003	0.358	0	0	0.004	0.290	-0.008	0.008	0.004	0.290
**Personal possession of N-95 mask**	0.030	0.061	0.001	0.621	-0.142	0.077	0.011	0.068	-0.160	0.092	0.010	0.082	-0.084	0.065	0.006	0.199	-1.849	1.435	0.006	0.199
**Perceived usefulness of N-95 mask**	0.045	0.133	0	0.733	0.173	0.167	0.004	0.300	0.230	0.198	0.005	0.247	0.142	0.141	0.003	0.314	3.130	3.102	0.003	0.314
**Total number of physical symptoms**	0.048	0.011	0.061	<0.001	0.075	0.013	0.095	<0.001	0.130	0.015	0.207	<0.001	0.080	0.011	0.153	<0.001	1.759	0.240	0.153	<0.001

IES-R = Impact of Event Scale - Revised
